# Effect of aromatase inhibitors for preventing ovarian hyperstimulation syndrome in infertile patients undergoing in vitro fertilization: a systematic review and meta-analysis

**DOI:** 10.1186/s12958-024-01258-y

**Published:** 2024-07-23

**Authors:** Linying Jiang, Yuhan Qiu, Lijuan Xu, Ruiqi Chang, Fan He

**Affiliations:** 1 Department of Obstetrics and Gynecology, The Second Affiliated Hospital of Chongqing Medical University, Chongqing, People’s Republic of China; 2 The Center for Reproductive Medicine, Department of Obstetrics and Gynecology, The Second Affiliated Hospital of Chongqing Medical University, Chongqing, People’s Republic of China; 3 Joint International Research Lab for Reproduction and Development, Ministry of Education, Chongqing, People’s Republic of China; 4 Reproduction and Stem Cell Therapy Research Center of Chongqing, Chongqing, People’s Republic of China

**Keywords:** Aromatase inhibitors, Letrozole, Ovarian hyperstimulation syndrome, In vitro fertilization, Systematic review, Meta-analysis

## Abstract

**Purpose:**

To summarize the findings of relevant randomized controlled trials (RCTs) and conduct a meta-analysis to investigate the potential effect of aromatase inhibitors on preventing moderate to severe ovarian hyperstimulation syndrome (OHSS) in infertile women undergoing in vitro fertilization (IVF).

**Methods:**

We searched for relevant RCTs in electronic databases, including MEDLINE, Embase, Cochrane Central Register of Controlled Trials (CENTRAL), and ClinicalTrials.gov (from inception to August 2023). In addition, we manually searched the related reviews and reference lists of included studies for further relevant studies. We included RCTs where aromatase inhibitors prescribed either during controlled ovarian stimulation (COS) or in early luteal phase. The meta-analysis was performed using RevMan 5.4.1 software. The primary outcome was the incidence of moderate to severe OHSS. A descriptive analysis was conducted in cases where a meta-analysis was not feasible due to heterogeneity or lack of comparable data.

**Results:**

2858 records were retrieved and 12 RCTs were finally included. Letrozole was administered in the treatment group during COS in seven RCTs, whereas in the early luteal phase in five RCTs. Compared with the control group, the risk of moderate to severe OHSS significantly reduced by 55% in the letrozole group (*RR* 0.45, 95% *CI* 0.32 to 0.64, *I*^*2*^ = 0%, 5 RCTs, 494 patients). Moreover, serum estradiol (E2) levels on hCG trigger day significantly decreased with the administration of letrozole during COS (*MD* -847.23, 95% *CI* -1398.00 to -296.47, *I*^*2*^ = 93%, 5 RCTs, 374 patients). And serum E2 levels on the 4th, 5th and 7th to 10th day after hCG trigger were also significantly lower than those in the control group when letrozole was administered in the early luteal phase.

**Conclusions:**

Patients with high risk of OHSS probably benefit from letrozole, which has been revealed to reduce the incidence of moderate to severe OHSS by this systematic review. However, the very limited number of participants and the quality of the included studies does not allow to recommend letrozole for the prevention of severe OHSS.

**Supplementary Information:**

The online version contains supplementary material available at 10.1186/s12958-024-01258-y.

## Introduction

Ovarian hyperstimulation syndrome (OHSS) is a common complication associated with controlled ovarian stimulation (COS) in in vitro fertilization (IVF) cycles. OHSS is characterized by the development of multiple follicles in bilateral ovaries, ovarian enlargement, increased vascular permeability and a fluid shift into the third space. OHSS can be classified as either early or late, based on its occurrence in the early luteal phase or early pregnancy, respectively [1]. Early OHSS typically occurs within nine days following the human chorionic gonadotropin (hCG) trigger [2]. According to clinical manifestations and laboratory findings, OHSS can be categorized as mild, moderate, severe, and critical [2, 3]. Although OHSS is self-limiting and usually alleviates within two weeks, moderate to severe OHSS can lead to serious clinical complications that necessitates hospitalization, such as ascites, pleural effusion, renal function injury, electrolyte disturbances, hypercoagulability and thrombosis [3, 4]. Furthermore, it can be life-threatening and even fatal in more serious or critical cases [5, 6]. In IVF cycles, the estimated incidence of moderate to severe OHSS ranges from 3 to 8% [7, 8]. Therefore, how to prevent moderate to severe OHSS is of essential importance.

Early OHSS is associated with the administration of hCG trigger in COS. An acute surge in hCG levels stimulates the granulosa-lutein cells to produce vascular endothelial growth factor (VEGF), which is responsible for increasing vascular permeability [9]. In addition, high serum estradiol (E2) levels during COS are also associated with the occurrence of OHSS. In fact, there is ample evidence that the risk of OHSS significantly increases when serum E2 concentration is greater than 3500 pg/mL [3, 10]. One possibility is that estrogens upregulate the expression of cyclic adenosine monophosphate (cAMP), which further activates the cAMP-dependent protein kinase A, and ultimately promotes the transcription of VEGF [11]. Another possibility is that high E2 levels may upregulate the expression of cystic fibrosis transmembrane conductance regulator (CFTR) and aquaporin 1 (AQP1) in peritoneal epithelial cells [12]. The synergistic effect of CFTR and AQP1 plays an important role in the process of peritoneal fluid effusion and accumulation [12, 13]. Hence, inhibiting excessive E2 production, either during COS or in the early luteal phase, may serve as an effective measure to prevent OHSS.

Aromatase inhibitors, with letrozole being one of the most frequently used, are commonly administered for ovulation induction. Letrozole works by blocking the conversion of androgens to estrogens through the inhibition of aromatase activity, subsequently reducing the secretion of ovarian estrogen. Administering aromatase inhibitors during COS had been shown to enhance ovarian response, reduce required gonadotropin doses, and decrease estrogen levels [14, 15]. Moreover, taking letrozole after oocyte retrieval has been found to potentially prevent OHSS by downregulating estrogen and VEGF levels [16]. A systematic review published in 2020 showed that there was a significant decrease in the incidence of total OHSS and moderate to severe OHSS with the administration of letrozole [17]. However, this systematic review included both retrospective and prospective studies, the former of which have a higher risk of bias. In addition, studies using letrozole in the follicular phase were not included in this systematic review. Most importantly, six randomized controlled trials (RCTs) have been published in the past three years [14, 15, 18–21]. In this systematic review, we aim to only include RCTs and assess the risk of OHSS following treatment with aromatase inhibitors, either during COS or in the early luteal phase, in IVF cycles.

## Methods

### Search strategy

We conducted a comprehensive electronic search of MEDLINE, Embase, Cochrane Central Register of Controlled Trials (CENTRAL), and ClinicalTrials.gov databases, spanning from their inception to August, 2023. Our search strategy incorporated subject heading terms, text terms with truncation ($), and proximity operators (NEAR and ADJ) as appropriate: ‘ovarian hyperstimulation syndrome’, ‘OHSS’, ‘aromatase inhibitors’, ‘Aminoglutethimide’, ‘Fadrozole’, ‘Anastrozole’, ‘Arimidex’, ‘Letrozole’, ‘Femara’, ‘Exemestane’, ‘Aromasin’, ‘Vorozole’, ‘Rivizor’, ‘Formestane’, ‘Lentaron’, ‘Afema’, ‘randomized controlled trial’, etc. We also screened the reference lists of relevant literature to identify additional potentially eligible trials. The literature search was independently performed by two reviewers (LYJ and YHQ). The electronic search strategies are presented in Appendix 1.

### Study selection

The following inclusion criteria were applied: (1) RCTs on aromatase inhibitors prescribed during COS or in early luteal phase in IVF cycles; (2) comparisons involving different aromatase inhibitors, or comparisons of treatment with aromatase inhibitors against controls, which includes placebo, no treatment or other drugs; (3) published in English language. RCTs that recruited patients with either diminished ovarian reserve or poor ovarian response were excluded. Two reviewers (LYJ and YHQ) independently reviewed the titles and abstracts, and obtained the full texts for all studies that appeared potentially eligible. Any disagreements between the two reviewers were resolved through discussion or by consulting a third reviewer (RQC).

### Data extraction

Two reviewers (LYJ and YHQ) independently extracted data, including study characteristics, methodological details, and outcome data. Discrepancies were resolved through consensus or by consulting a third reviewer (RQC).

### Risk of bias in included studies

Using the Cochrane Risk of Bias Tool, two reviewers (LYJ and YHQ) independently assessed the risk of bias across the following domains: random sequence generation, allocation concealment, blinding of participants and personnel, blinding of outcome assessment, incomplete outcome data, selective reporting, and other potential biases [22]. The risk of bias was rated as ‘low’, ‘unclear’ or ‘high’. Any disagreements were resolved by discussion or arbitration from a third reviewer (RQC).

### Statistical analysis

The primary outcome is the incidence of moderate to severe OHSS. Secondary outcomes include the overall incidence of OHSS, the incidence of mild, moderate, severe and critical OHSS, and serum E2 levels. Meta-analyses were performed using Review Manager (RevMan) 5.4.1 software. The risk ratio (*RR*) with corresponding 95% confidence intervals (*CI*s) was used for dichotomous variables, and continuous data were expressed as mean difference (*MD*) with 95% *CI*. The heterogeneity between the results of different studies was assessed using the *CHI*^*2*^ test and *I*^*2*^ statistics. Results were judged as low heterogeneity (*I*^*2*^ < 25%), medium heterogeneity (*I*^*2*^ = 25–50%), or high heterogeneity (*I*^*2*^ > 50%), respectively. Substantial heterogeneity (*I*^*2*^ > 50%) was addressed by (1) rechecking the data for accuracy; (2) conducting subgroup analyses; (3) excluding studies deemed to have a high risk of bias. The random-effects model was applied to cases of substantial heterogeneity that remained unexplained. In all other instances, the fixed-effects model was utilized. The results were presented by forest plots, and *P*-values < 0.05 were considered significantly different. Pre-specified subgroup analyses were performed based on the medication time (during COS or in early luteal phase). The descriptive analysis was conducted when a meta-analysis was not possible due to few included studies or high clinical heterogeneity.

## Results

### Search results

**Fig. 1:**
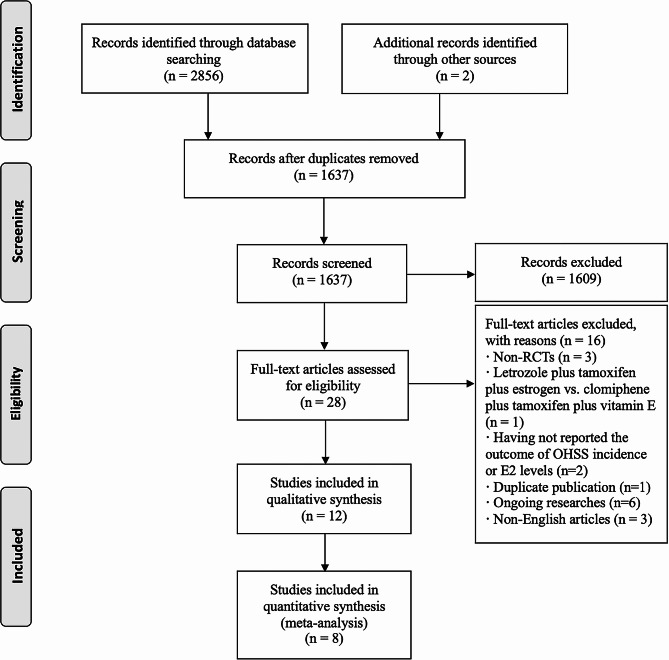
PRISMA study flow diagram

From the search, we obtained 2858 citations, with 1221 identified as duplicates. Upon screening titles and abstracts, we excluded 1609 articles, retaining 28 for a full-text review. Out of these, 12 RCTs met our eligibility criteria and were included in this systematic review [14–16, 18–21, 23–27]. Of these, eight were subjected to quantitative analysis [15, 16, 18, 19, 21, 25–27]. Qualitative analysis was performed for four RCTs. Three RCTs did not report the incidence of OHSS and serum E2 levels which could be pooled into meta-analysis and the authors have not replied to our emails [14, 23, 24]. Another RCT compared the efficiency of letrozole for the prevention of OHSS with ganirelix acetate [20]. A flow chart illustrating the search and selection process is displayed in Fig. 1.

### Characteristics of included studies

The twelve RCTs comprised 569 participants in the treatment group and another 524 in the control group. Letrozole was administered in the treatment group during COS in seven RCTs [14, 15, 18, 19, 21, 25, 27], whereas in the early luteal phase in five RCTs [16, 20, 23, 24, 26]. The participants were treated with gonadotrophin-releasing hormone (GnRH) antagonist protocol in five RCTs [14, 18, 19, 21, 25], GnRH agonist long protocol in six RCTs [15, 16, 20, 23, 24, 27], and GnRH agonist protocol in one RCT [26]. The freeze-all and fresh transfer strategy was used in seven RCTs [16, 19–21, 23, 24, 26] and five RCTs [14, 15, 18, 25, 27], respectively. Eight RCTs reported the incidence of OHSS [16, 18–21, 25–27]. Five RCTs utilized diagnostic criteria for OHSS [4, 8, 28, 29] that were internationally recognized, albeit with slightly variations [16, 19–21, 26]. However, three RCTs did not describe the criteria for OHSS diagnosis [18, 25, 27]. Six RCTs investigated the efficacy of letrozole in preventing moderate to severe OHSS and specifically severe OHSS [16, 19–21, 25, 26]. Among them, four RCTs compared the incidence of OHSS in patients treated with letrozole versus placebo or no treatment [16, 19, 21, 25]; one RCT compared letrozole with aspirin, which was considered as a placebo [26]; and the other one RCT evaluated the effect of letrozole versus ganirelix acetate (gonadotropin-releasing hormone antagonist) in OHSS prevention [20]. Five RCTs reported the incidence of mild OHSS after letrozole treatment [19–21, 25, 26]. Serum E2 levels were evaluated in all RCTs. Seven RCTs used letrozole during COS, and all of them reported serum E2 levels on hCG trigger day [14, 15, 18, 19, 21, 25, 27]. Five RCTs used letrozole in early luteal phase [16, 20, 23, 24, 26], among them, two RCTs reported serum E2 levels on the 4th, 7th and 10th day after hCG trigger [23, 24]; one RCT reported serum E2 levels on the 7th day after hCG trigger [26]; one RCT displayed the trend of serum E2 levels on the 5th, 8th and 10th day after hCG trigger without reporting specific E2 values [16]; and one RCT reported serum E2 levels on the 5th and 7th day after oocyte retrieval [20]. The characteristics of the included studies are summarized in Table 1.

**Table 1:** Characteristics of the included studies

Study	Study population	Sample size (T/C)	Standard for OHSS diagnosis	Intervention time	Treatment	Control	COS protocol	Strategy of embryo transfer	Overall OHSS (T/C)	Mild OHSS (T/C)	Moderate OHSS (T/C)	Severe OHSS (T/C)	E2 levels on hCG trigger day (T vs. C, pg/ml)	E2 levels in luteal phase (T vs. C, pg/ml)
Fatemi 2008 [23]	Oocyte donors	3/3	N.A.	After OR	Letrozole 2.5 mg bid until menstruation, for a maximum of 14 days	Placebo	GnRH-a long	Frozen	N.A.	N.A.	N.A.	N.A.	3580.67 ± 817.2 vs. 2584 ± 779.5 (NS)	On 4th, 7th and 10th day after hCG trigger: 272 ± 65.4 vs. 749 ± 27.4 (4th, *P* = 0.008) 229 ± 69 vs. 1457 ± 152 (7th, *P* = 0.005) 31 ± 7 vs. 1308 ± 88 (10th, *P* = 0.004)
Garcia-Velasco 2009 [24]	Oocyte donors	15/15	N.A.	After OR	Letrozole 2.5 mg qd, for 5 days	Placebo	GnRH-a long	Frozen	N.A.	N.A.	N.A.	N.A.	1858 vs. 2143 (*P* = 0.24)	On 4th, 7th and 10th day after hCG trigger: 279 vs. 1586 (4th, *P* < 0.001) 240 vs. 855 (7th, *P* < 0.001) 40 vs. 448 (10th, *P* < 0.001)
Mukherjee 2012 [25]	Severe male factor infertility	42/52	N.A.	During COS	Letrozole 5 mg qd from SD-2, for 5 days	No treatment	GnRH-ant	Fresh	0/7	0/5	0/2	0/0	830 ± 36 vs. 1076 ± 41 (*P* < 0.001)	Not detected
He 2014 [16]	Participants with high risk of OHSS	64/24	Golan^[28]^	After OR	Letrozole 2.5 mg qd /bid/tid, for 5 days	Placebo	GnRH-a long	Frozen	9/9	N.A.	9/8	0/1	7840.34 ± 1734.73 vs. 7835.41 ± 1740.5 (*P* = 0.44)	On 5th, 8th and 10th day after hCG trigger: significantly lower in letrozole group (as shown by the figure)
Mai 2017 [26]	Participants with high risk of OHSS	112/102	Navot^[29]^	After OR	Letrozole 2.5 mg bid, for 5 days	Aspirin 100 mg qd, for 5 days	GnRH-a	Frozen	90/92	62/46	22/36	6/10	Not detected	On 7th day after hCG trigger: 84.0 (15.0-223.5) vs. 3110.5 (113.3-4976.8) (*P* < 0.001)
Yang 2019 [27]	Participants with high ovarian response	65/65	N.A.	During COS	Letrozole 2.5 mg qd from SD + 4 to the trigger day	No treatment	GnRH-a long	Fresh	0/1	N.A.	N.A.	N.A.	1404.28 (431.3, 6227) vs. 2867 (1043.62, 7198) (*P* < 0.001)	Not detected
Eftekhar 2020 [18]	Participants with normal ovarian response	50/50	N.A.	During COS	Letrozole 5 mg qd from SD to the trigger day	No treatment	GnRH-ant	Fresh	2/2	N.A.	N.A.	N.A.	311.04 ± 229.64 vs. 1350.99 ± 833.29 (*P* < 0.001)	Not detected
Tshzmachyan 2020 [19]	Participants with polycystic ovary syndrome	24/24	RCOG Green-top Guideline No. 5^[8]^	During COS	Letrozole 5 mg qd from SD-1, for 5 days	No treatment	GnRH-ant	Frozen	2/10	2/9	0/1	0/0	2526.58 ± 1330.0 vs. 4412.21 ± 2568.3 (*P* = 0.003)	Not detected
Choudhary 2021 [20]	Participants with high risk of OHSS	61/61	Namavar^[4]^	After OR	Letrozole 2.5 mg bid, for 7 days	Ganirelix Acetate 0.25 mg qd, for 7 days	GnRH-a long	Frozen	8/12	0/0	6/10	2/2	5896.38 ± 2240.04 vs. 5052.38 ± 2211.887 (*P* = 0.035)	On 5th and 7th day after oocyte retrieval: 685.74 ± 1066.55 (7th, *P* = 0.001) 2364.82 ± 1774.25 (9th, *P* = 0.001)
Bülow 2022 [14]	Participants with normal ovarian response	67/62	N.A.	During COS	Letrozole 5 mg qd from SD until the day before the trigger day	Placebo	GnRH-ant	Fresh	N.A.	N.A.	N.A.	N.A.	366 (256–591) vs. 1176 (799–1682) (*P* < 0.001)	Luteal phase (AUC): 2145 (1138–3910) vs. 7138 (5153–9364) (*P* < 0.001)
Ebrahimi 2022 [15]	Infertile women with endometriosis	41/41	N.A.	During COS	Letrozole 5 mg qd from SD, for 5 days	Placebo	GnRH-a long	Fresh	N.A.	N.A.	N.A.	N.A.	1837.62 ± 1181.69 vs. 2604.51 ± 1823.36 (*P* = 0.02)	Not detected
Ghasemi Tehrani 2022 [21]	Participants with polycystic ovary syndrome	25/25	Golan^[28]^	During COS	Letrozole 5 mg qd from SD, for 5 days	Placebo	GnRH-ant	Frozen	7/13	6/4	1/9	0/0	4902.48 ± 1252.944 vs. 5744.92 ± 1621.78 (*P* = 0.04)	Not detected

*T* treatment group, *C* control group, *GnRH-a* gonadotrophin-releasing hormone agonist, *GnRH-ant* gonadotrophin-releasing hormone antagonist, *E2* estradiol, *OHSS* ovarian hyperstimulation syndrome, *COS* controlled ovarian stimulation, *OR* oocyte retrieval, *SD* stimulation day, *SD-2* two days before stimulation day, *SD-1* one day before stimulation day, *SD + 4* four days after stimulation day, *qd* once a day, *bid* twice a day, *tid* three times a day, vs. versus, *N.A*. not available, *NS* not significant

Values are mean ± standard deviation, median (min, max), median (interquartile range)

### Risk of bias in included studies

**Fig. 2:**
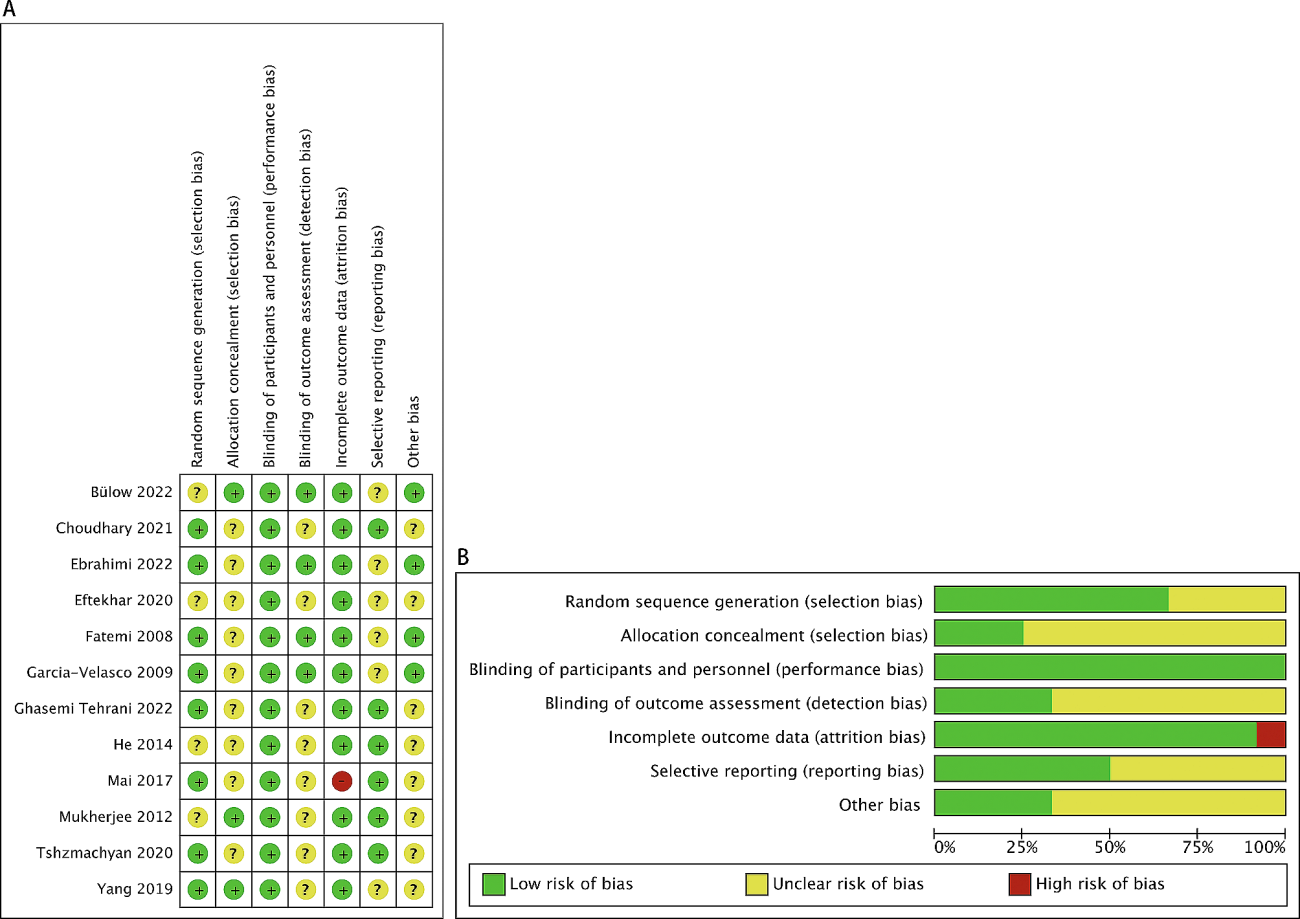
**A** Risk of bias graph: review authors’ judgements about each risk of bias item presented as percentages across all included studies and **B** Risk of bias summary: review authors’ judgements about each risk of bias item for each included study

We assessed the risk of bias for all included studies. In terms of performance bias and detection bias, we assessed the risk based on the reported outcome of OHSS incidence; if not reported, we evaluated the outcome of E2 levels. Eight RCTs provided clear methods for random sequence generation. They used methods like computer-generated randomization list, block randomization, or drawing lots, leading them to be rated as having a low risk of bias [15, 19–21, 23, 24, 26, 27]. The remaining four RCTs were judged as unclear risk due to lack of relative information [14, 16, 18, 25]. Three RCTs were at low risk of bias for allocation concealment as the sealed envelope method was used to randomly allocate patients into two groups [14, 25, 27]. Nine RCTs providing no detailed information were judged as unclear risk of allocation concealment [15, 16, 18–21, 23, 24, 26]. We consider that whether using blinding of participants and personnel was less likely to exert effects on any outcomes evaluated by this review, so all RCTs were assessed as low risk of bias in this domain [14–16, 18–21, 23–27]. Four RCTs had a low risk of detection bias, as they exclusively reported outcomes related to of E2 levels, determined through automated analysis [14, 15, 23, 24]. The rest eight RCTs reported the outcome of OHSS incidence involving subjective judgement, but these trials did not mention blinding of outcome assessors, hence they were at unclear risk of bias [16, 18–21, 25–27]. Nine RCTs did not reported any losses to follow-up [16, 18–21, 23–25, 27], and two RCTs, providing missing data and reasons for discontinuation, were balanced between groups [14, 15], therefore they were rated as low risk of attrition bias. The other one RCT was judged as high risk in this domain because it excluded more than 10% patients from the analysis and there was a clear difference in the proportion of missing between the treatment and control groups [26]. Six RCTs were at low risk of reporting bias due to the fact that they reported the incidence moderate to severe OHSS [16, 19–21, 25, 26]. Six RCTs did not reported this primary outcome, so they were considered as unclear risk [14, 15, 18, 23, 24, 27]. The other biases were assessed at last. In seven out of the eight RCTs which reported the incidence of OHSS, there were no significant differences in major baseline characteristics, including female age, body mass index (BMI), ovarian reserve markers (anti-Müllerian hormone, antral follicle count, baseline follicle stimulating hormone or the combination of two or three aforementioned markers), and number of oocytes retrieved between the letrozole and control groups [16, 19–21, 25–27]. Only one RCT was rated as unclear risk of this domain because the authors did not report BMI, number of oocytes retrieved or number of mature follicles and have not replied to our email [18]. In addition, three RCTs did not describe the diagnostic criteria of OHSS [18, 25, 27]; five RCTs recruited patients with high risk of OHSS, so there was a possible contamination bias [16, 19–21, 26]. Therefore, these RCTs are at unclear risk of other biases [16, 18–21, 25–27]. The assessment of the “risk of bias” based on Cochrane’s criteria is shown in Fig. 2A and B.

### Primary outcome

#### Incidence of moderate to severe OHSS

**Fig. 3:**
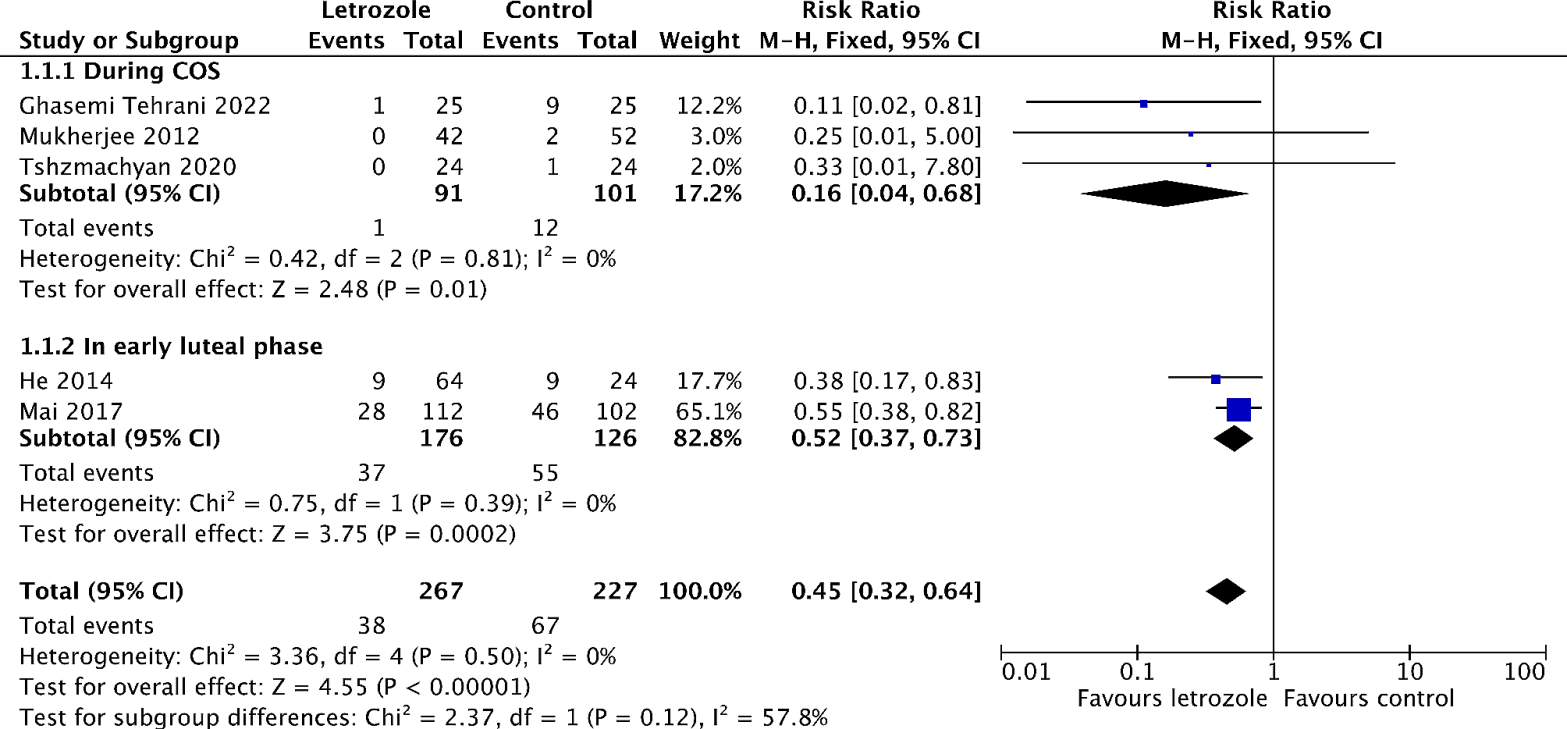
Forest plot of comparison: letrozole versus control, outcome: incidence of moderate to severe OHSS

**Fig. 4:**
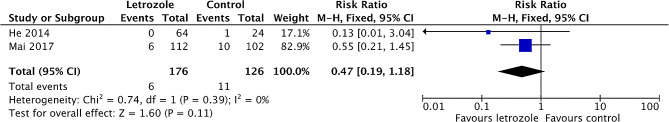
Forest plot of comparison: letrozole versus control, outcome: incidence of severe OHSS

Six studies reported on the incidence of moderate to severe OHSS [16, 19–21, 25, 26]. Out of these, five studies made comparison between letrozole and either a placebo or no treatment [16, 19, 21, 25, 26]. The meta-analysis showed that the risk of moderate to severe OHSS significantly reduced by 55% in the letrozole group (*RR* 0.45, 95% *CI* 0.32 to 0.64, *I*^*2*^ = 0%, 5 RCTs, 494 patients) (Fig. 3). The subgroup analysis revealed the risk of moderate to severe OHSS is lower when letrozole was administered during COS (*RR* 0.16, 95% *CI* 0.04 to 0.68, *I*^*2*^ = 0%, 3 RCTs, 192 patients), compared to in the early luteal phase (*RR* 0.52, 95% *CI* 0.37 to 0.73, *I*^*2*^ = 0%, 2 RCTs, 302 patients) (Fig. 3). The similar results were obtained when only RCTs recruiting participants with high risk of OHSS are pooled in meta-analysis (Figure S2). In addition, the risk of moderate OHSS significantly decreased by 55% in the letrozole group (*RR* 0.45, 95% *CI* 0.31 to 0.66, *I*^*2*^ = 0%, 5 RCTs, 494 patients) (Figure S3). Although not significant, there was a trend toward a lower risk of severe OHSS in the letrozole group (*RR* 0.47, 95% *CI* 0.19 to 1.18, *I*^*2*^ = 0%, 2 RCTs, 494 patients) (Fig. 4). One study has not been included in the meta-analysis as it compared the efficiency of letrozole for the prevention of OHSS with ganirelix acetate [20]. Letrozole might be more effective than ganirelix acetate in preventing moderate OHSS, although the difference of incidence was not statistically significant (9.8% vs. 16.3%, *P* = 0.38). Both drugs displayed equivalent efficacy in severe OHSS prevention (3.3 vs. 3.3%, *P* = 1.00).

### Secondary outcomes

#### Incidence of overall OHSS

**Fig. 5:**
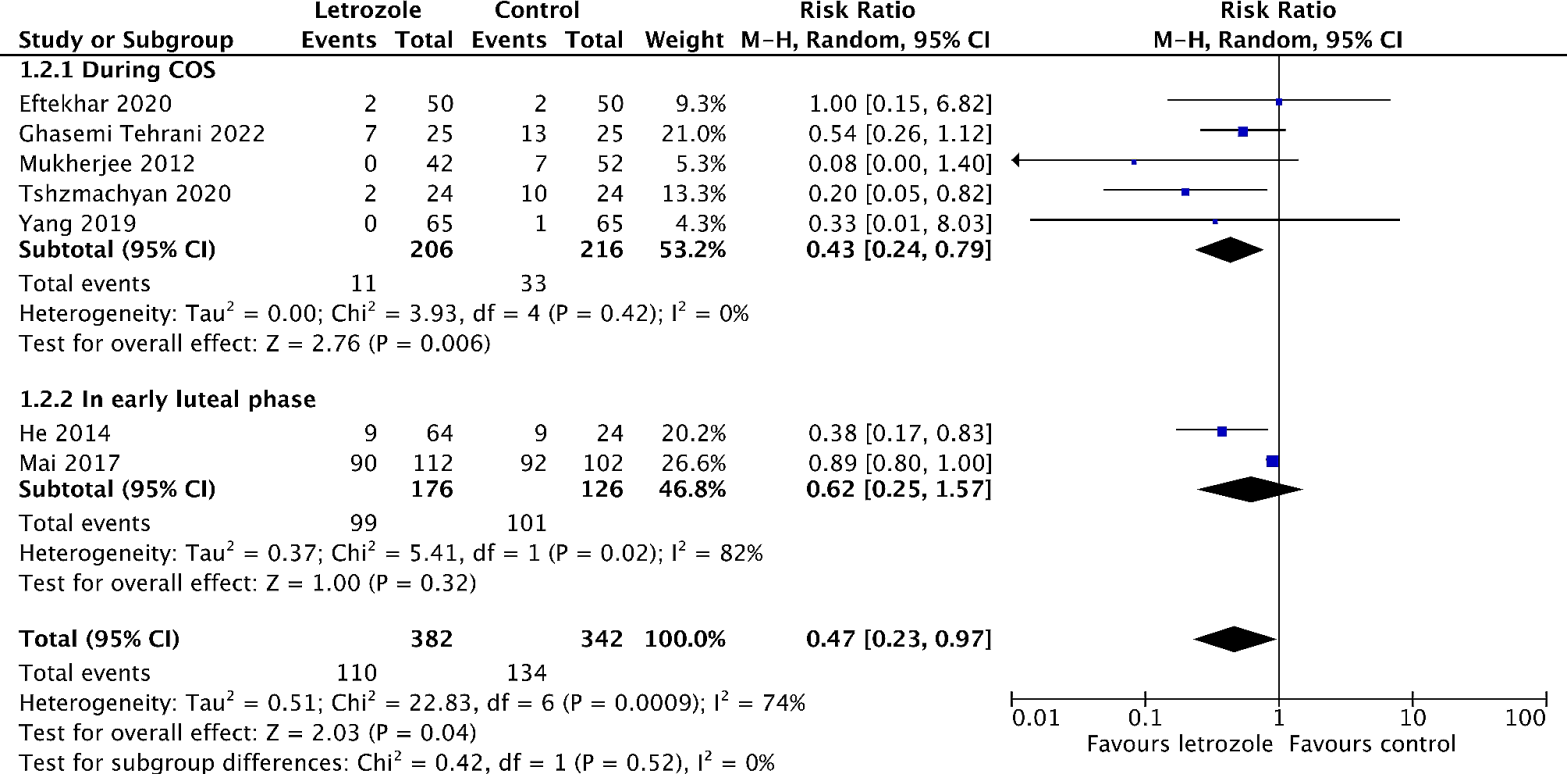
Forest plot of comparison: letrozole versus control, outcome: incidence of overall OHSS

Eight studies reported on the incidence of overall OHSS [16, 18–21, 25–27]. Of these, seven studies made a comparison between letrozole and either a placebo or no treatment [16, 18, 19, 21, 25–27]. The meta-analysis showed that the risk of overall OHSS significantly reduced by 53% in the letrozole group (*RR* 0.47, 95% *CI* 0.23 to 0.97, *I*^*2*^ = 74%, 7 RCTs, 724 patients) (Fig. 5). Notably, the subgroup analysis revealed that only when letrozole was administered during COS, the risk of overall OHSS significantly reduced(*RR* 0.43, 95% *CI* 0.24 to 0.79, *I*^*2*^ = 0%, 5 RCTs, 422 patients) (Fig. 5). Another study reported a reduced incidence of overall OHSS in the letrozole group when compared to that in the ganirelix acetate group (13.1% vs. 19.6%, *P* = 0.33) [20].

#### Incidence of mild OHSS

**Fig. 6:**
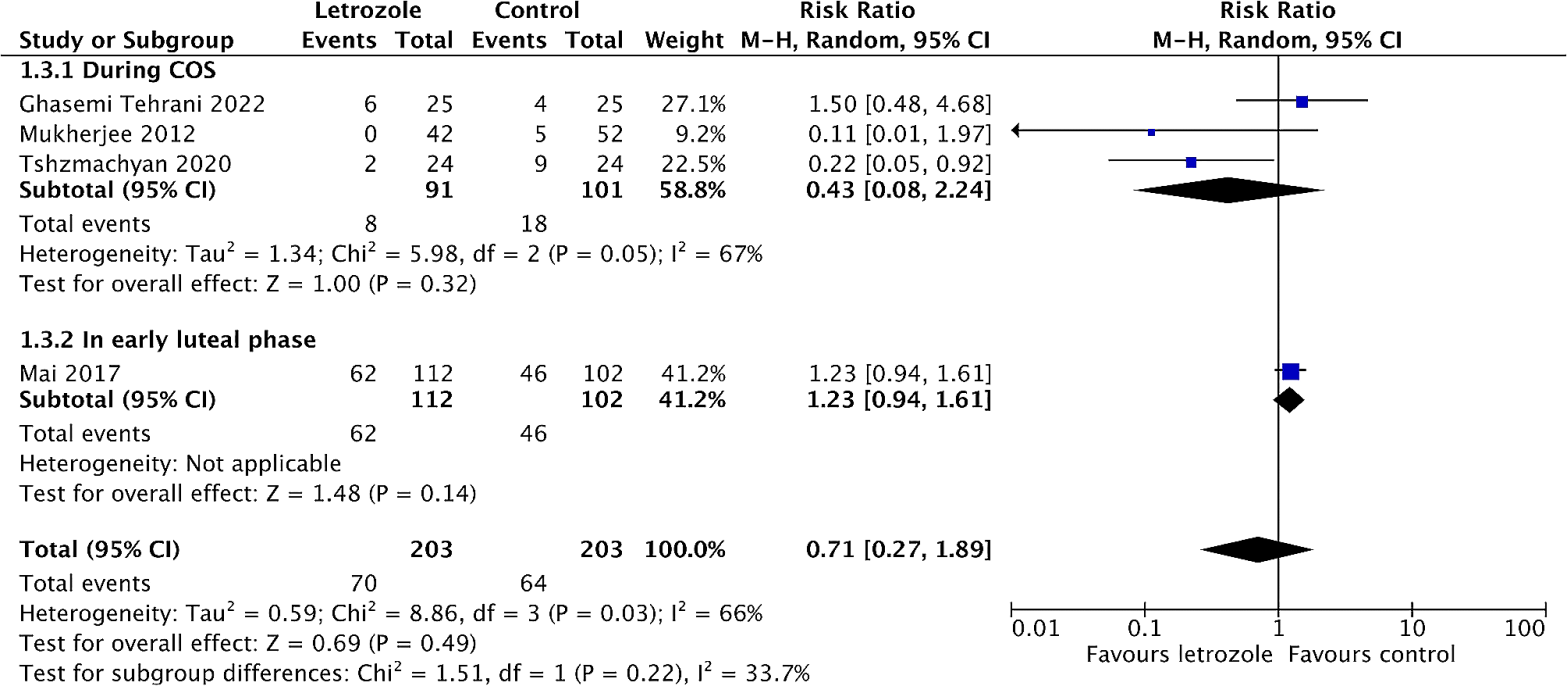
Forest plot of comparison: letrozole versus control, outcome: incidence of mild OHSS

Five studies reported on the incidence of mild OHSS [19–21, 25, 26], and four of them compared letrozole with either placebo or no treatment [19, 21, 25, 26]. The meta-analysis showed that there is a trend toward lower risk of mild OHSS with the administration of letrozole, although not significant (*RR* 0.71, 95% *CI* 0.27 to 1.89, *I*^*2*^ = 66%, 4 RCTs, 406 patients) (Fig. 6). Another study was excluded from meta-analysis as the treatment and control groups received letrozole and ganirelix acetate, respectively [20]. And none of mild OHSS cases were reported in this study.

#### Incidence of critical OHSS

Two studies reported on the incidence of critical OHSS [19, 26]. There were no cases of critical OHSS in both the treatment and control groups of these two studies.

### Serum E2 levels

**Fig. 7:**
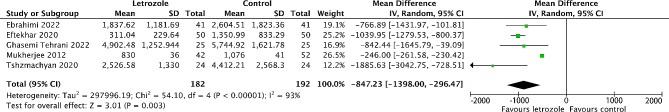
Forest plot of comparison: letrozole versus control, outcome: serum E2 levels on hCG trigger day

All twelve studies reported on serum E2 levels [14–16, 18–21, 23–27]. Seven studies reported serum E2 levels on hCG trigger day with the administration of letrozole during COS [14, 15, 18, 19, 21, 25, 27]. However, two of them were excluded from the meta-analysis because they did not present the outcome in the format of mean ± standard deviation [14, 27]. The meta-analysis showed that serum E2 levels on hCG trigger day were significantly lower in the treatment group (*MD* -847.23, 95% *CI* -1398.00 to -296.47, *I*^*2*^ = 93%, 5 RCTs, 374 patients) (Fig. 7). Five studies used letrozole in the early luteal phase [16, 20, 23, 24, 26]. Among them, two studies showed that serum E2 levels on the 4th (272 ± 65.4 vs. 749 ± 27.4 pg/ml and 279 vs. 1586 pg/ml), 7th (229 ± 69 vs. 1457 ± 152 pg/ml and 240 vs. 855 pg/ml) and 10th (31 ± 7 vs. 1308 ± 88 pg/ml and 40 vs. 448 pg/ml) day after hCG trigger significantly decreased in the letrozole group [23, 24]. However, the meta-analysis was not conducted as one of these two studies did not present the outcome as mean ± standard deviation [24]. Moreover, another study also reported that serum E2 levels on the 7th day after hCG trigger were lower in the letrozole group than those in the control group [84.0 (15.0-223.5) vs. 3110.5 (113.3-4976.8) pg/ml] [26]. One study reported that there was a significant trend toward lower E2 levels in the letrozole group on the 5th, 8th and 10th day after hCG trigger without reporting specific E2 values (*P* < 0.05) [16]. In addition, similar to the above results, one study found that serum E2 levels decreased on the 5th (685.74 ± 1066.55 vs. 2364.82 ± 1774.25 pg/ml) and 7th (218.38 ± 308.41 vs. 819.67 ± 848.14 pg/ml) day after oocyte retrieval when letrozole was compared with ganirelix acetate [20].

## Discussion

In this systematic review, we included 12 RCTs involving the letrozole administration during IVF cycles. Out of the 12 RCTs, 8 RCTs reported the incidence of OHSS, and 7 of these were included in meta-analysis. Our results suggest that letrozole significantly reduced the incidence of moderate and moderate to severe OHSS, whenever it was used during COS or in early luteal phase. In addition, the preventive effect of letrozole prescribed during COS was superior to that in early luteal phase. A previous systematic review only included studies in which letrozole administered in early luteal phase and also concluded that taking letrozole after oocyte retrieval can prevent moderate to severe OHSS [17]. Although not significant, our meta-analysis has revealed a trend toward a lower incidence of severe OHSS in the letrozole group compared with the control group. In Mai’s study [26], aspirin was used in the control group, and aspirin itself had been documented to reduce the incidence of severe OHSS [30, 31]. Therefore, the preventive effect of letrozole on severe OHSS might be underestimated. In any case, due to the small sample size of the two RCTs pooled in meta-analysis, RCTs with adequate power are needed to determine the efficacy of letrozole on severe OHSS prevention. As for the incidence of mild OHSS, no significantly decreasing effect has been found with the administration of letrozole, which is consistent with the previous systematic review [17]. Additionally, significantly lower serum E2 levels on the day of hCG administration and in luteal phase were associated with the prescription of letrozole during COS. And serum E2 levels during luteal phase in the letrozole group were also significantly lower than those in the control group when dosing letrozole after oocyte retrieval. It is noteworthy that one RCT [16] reported significantly lower serum VEGF levels in the letrozole group than those in the placebo group.

Several prior studies have highlighted the role of VEGF as a crucial factor in the development of OHSS. By regulating vascular permeability, VEGF facilitates thrombosis formation, which may subsequently promote the incidence of OHSS in the luteal phase [9, 32]. An RCT included in our systematic review found that serum VEGF levels in the letrozole group were significantly lower compared to the control group, and letrozole decreased VEGF production in a dose-dependent manner [16]. Therefore, letrozole administration after oocyte retrieval may prevent the occurrence of OHSS by reducing serum VEGF levels.

Estradiol undergoes metabolism through several pathways, including hydroxylation, methylation, glucuronidation, and sulfonation, resulting in the formation of estrogen metabolites (EMS) [33]. Specific estrogen metabolites, like 16-ketoestradiol and 4-hydroxyestrone, have been identified as stimulants for VEGF secretion in human luteinized granulosa cells. Moreover, along with the lowered level of E2 in human luteal tissues during the late luteal phase, the levels of both aforementioned EMS decreased significantly [34]. Furthermore, a decrease in plasma E2 levels in the late luteal phase correlates with an increase in 2-methoxyestradiol, an Ems, within the luteal tissue. Notably, this particular EMS has been shown to inhibit VEGF synthesis in human luteinized granulosa cells [35]. Collectively, the use of letrozole in the early luteal stage is speculated to reduce the levels of 16-ketoestradiol and 4-hydroxyestrone, both of which promote VEGF secretion, and up-regulate the level of 2-methoxyestradiol, which inhibit VEGF synthesis, through inhibiting the synthesis of estradiol in luteal tissues. Thus, OHSS can be prevented or alleviated due to the lowered level of VEGF.

Recent studies indicated that in OHSS model rats, letrozole treatment not only reduces serum E2 level and the diameter of the corpus luteum but also up-regulates the expression of caspase-3 and cleaved caspase-3 in ovarian tissues [36]. Furthermore, caspase-3 mediated apoptosis plays a key role in the regression of corpus luteum [37, 38]. Thus, by inducing apoptosis in luteal cells, letrozole might facilitate the regression of the corpus luteum (CL) and subsequently reduce the release of cytokines from luteal tissues that contribute to OHSS development. However, this hypothesis has not yet been verified in human luteal tissues.

Our systematic review of RCTs explored the use of letrozole for OHSS prevention, and offered valuable insights for its clinical prescription. In order to minimize the risk of bias during the systematic evaluation process, the standard Cochrane evaluation method was used to perform this study. Two reviewers independently handled literature retrieval, screening, and data extraction. A consensus was reached on discrepancies through team discussion or consulting a third reviewer. However, potential biases, notably publication bias and other reporting biases, remained to reduce the risk of publication bias, reviewers performed a comprehensive electronic search and a manual search in accordance with the Cochrane Manual, but omissions were still possible, especially for unpublished studies, non-English published studies, and grey literature. Therefore, the possibility of publication bias exists to some extent. However, reviewers did not assess publication bias using funnel plots as the number of included studies that reported the primary outcome was less than 10.

## Conclusion

Patients with high risk of OHSS probably benefit from letrozole, which has been revealed to reduce the incidence of moderate to severe OHSS by this systematic review. And a lower incidence of moderate to severe OHSS has been found with administering letrozole during COS, when compared to in the early luteal phase. Letrozole can prevent moderate to severe OHSS probably through decreasing serum E2 and VEGF levels. However, the very limited number of participants and the quality of the included studies does not allow to recommend letrozole for the prevention of severe OHSS. Further RCTs with high quality are urgently needed to further evaluate the efficacy of letrozole in the prevention of severe OHSS.

### Electronic supplementary material

Below is the link to the electronic supplementary material.

Supplementary Material 1

Supplementary Material 2

Supplementary Material 3

## Data Availability

No datasets were generated or analysed during the current study.
