# Supplementary material for: Effect of aromatase inhibitors for preventing ovarian hyperstimulation syndrome in infertile patients undergoing in vitro fertilization: a systematic review and meta-analysis
Source: Reprod Biol Endocrinol. 2024 Jul 23;22:85. doi: 10.1186/s12958-024-01258-y (PMC11265326; doi:10.1186/s12958-024-01258-y)
Supplement: Supplementary file 1 — Supplementary Material 1 [file 12958_2024_1258_MOESM1_ESM.docx]

**Supplementary Appendix 1: Details of Search Strategy**
**Database:**

Ovid MEDLINE(R) ALL <1946 to August 31, 2023>

| **#** | **Query** |
| --- | --- |
| 1 | exp Ovulation induction/ or exp Superovulation/ |
| 2 | (ovulat$ adj3 induc$).tw. |
| 3 | superovulation.tw. |
| 4 | (ovari$ adj2 hyperstimulat$).tw. |
| 5 | (ovari$ adj2 stimulat$).tw. |
| 6 | exp Ovarian Hyperstimulation Syndrome/ |
| 7 | (ovari$ adj3 syndrome$).tw. |
| 8 | OHSS.tw. |
| 9 | exp Embryo transfer/ or exp Fertilization in vitro/ or exp Sperm injections intracytoplasmic/ |
| 10 | (ET or IVF or ICSI).tw. |
| 11 | (embryo$ adj2 transfer$).tw. |
| 12 | (blastocyst$ adj2 transfer$).tw. |
| 13 | vitro fertili?ation.tw. |
| 14 | intracytoplasmic sperm injection$.tw. |
| 15 | 1 or 2 or 3 or 4 or 5 or 6 or 7 or 8 or 9 or 10 or 11 or 12 or 13 or 14 |
| 16 | exp Aromatase inhibitors/ or exp Aminoglutethimide/ or exp Fadrozole/ |
| 17 | aromatase inhibitor$.tw. |
| 18 | aminoglutethimide.tw. |
| 19 | (Anastrozole or Arimidex or Letrozole or Femara or Exemestane or Aromasin or Vorozole or Rivizor or Formestane or Lentaron or Fadrozole or Afema).tw. |
| 20 | 16 or 17 or 18 or 19 |
| 21 | 15 and 20 |
| 22 | randomized controlled trial.pt. |
| 23 | controlled clinical trial.pt. |
| 24 | randomized.ab. |
| 25 | placebo.ab. |
| 26 | randomly.ab. |
| 27 | trial.ti. |
| 28 | clinical trials as topic.sh. |
| 29 | 22 or 23 or 24 or 25 or 26 or 27 or 28 |
| 30 | exp animals/ not humans.sh. |
| 31 | 29 not 30 |
| 32 | 21 and 31 |

**Database:**

Embase <1974 to 2023 August 31>

| **#** | **Query** |
| --- | --- |
| 1 | exp ovarian hyperstimulation syndrome/ or exp ovulation induction/ or exp superovulation/ |
| 2 | (ovari$ adj3 syndrome$).tw. |
| 3 | OHSS.tw. |
| 4 | (ovulat$ adj3 induc$).tw. |
| 5 | superovulation.tw. |
| 6 | (ovari$ adj3 hyperstimulat$).tw. |
| 7 | (ovari$ adj3 stimulat$).tw. |
| 8 | COH.tw. |
| 9 | exp embryo transfer/ or exp fertilization in vitro/ or exp intracytoplasmic sperm injections/ |
| 10 | (ET or IVF or ICSI).tw. |
| 11 | (embryo$ adj3 transfer$).tw. |
| 12 | (blastocyst$ adj3 transfer$).tw. |
| 13 | vitro fertili?ation.tw. |
| 14 | intracytoplasmic sperm injection$.tw. |
| 15 | 1 or 2 or 3 or 4 or 5 or 6 or 7 or 8 or 9 or 10 or 11 or 12 or 13 or 14 |
| 16 | exp aromatase inhibitor/ or exp letrozole/ or exp exemestane/ or exp vorozole/ or exp anastrozole/ or exp fadrozole/ |
| 17 | exp aminoglutethimide/ |
| 18 | aromatase inhibitor$.tw. |
| 19 | aminoglutethimide.tw. |
| 20 | (Anastrozole or Arimidex or Letrozole or Femara or Exemestane or Aromasin or Vorozole or Rivizor or Formestane or Lentaron or Fadrozole or Afema).tw. |
| 21 | 16 or 17 or 18 or 19 or 20 |
| 22 | 15 and 21 |
| 23 | exp randomized controlled trial/ |
| 24 | exp double blind procedure/ |
| 25 | exp single blind procedure/ |
| 26 | exp crossover procedure/ |
| 27 | (random$ or factorial$ or placebo$ or assign$ or allocat$ or volunteer$ or crossover$ or cross-over$).tw. |
| 28 | (doubl$ adj blind$).tw. |
| 29 | (singl$ adj blind$).tw. |
| 30 | 23 or 24 or 25 or 26 or 27 or 28 or 29 |
| 31 | ((animal not (human and animal)).sh. |
| 32 | 30 not 31 |
| 33 | 22 and 32 |

**Database:**

EBM Reviews - Cochrane Central Register of Controlled Trials < August 2023>

| **#** | **Query** |
| --- | --- |
| 1 | exp ovarian hyperstimulation syndrome/ or exp ovulation induction/ or exp superovulation/ |
| 2 | (ovari$ adj3 syndrome$).tw. |
| 3 | OHSS.tw. |
| 4 | superovulation.tw. |
| 5 | (ovulat$ adj3 induc$).tw. |
| 6 | (ovari$ adj3 hyperstimulat$).tw. |
| 7 | (ovari$ adj3 stimulat$).tw. |
| 8 | COH.tw. |
| 9 | exp embryo transfer/ or exp fertilization in vitro/ or exp Sperm Injections, Intracytoplasmic/ |
| 10 | (ET or IVF or ICSI).tw. |
| 11 | (embryo$ adj3 transfer$).tw. |
| 12 | (blastocyst$ adj3 transfer$).tw. |
| 13 | vitro fertili?ation.tw. |
| 14 | intracytoplasmic sperm injection$.tw. |
| 15 | 1 or 2 or 3 or 4 or 5 or 6 or 7 or 8 or 9 or 10 or 11 or 12 or 13 or 14 |
| 16 | exp Aromatase Inhibitors/ or exp fadrozole/ or exp aromatase/ |
| 17 | exp aminoglutethimide/ |
| 18 | aromatase inhibitor$.tw. |
| 19 | aminoglutethimide.tw. |
| 20 | (Anastrozole or Arimidex or Letrozole or Femara or Exemestane or Aromasin or Vorozole or Rivizor or FormestaneorLentaron or Fadrozole or Afema).tw. |
| 21 | 16 or 17 or 18 or 19 or 20 |
| 22 | 15 and 21 |
