# Supplementary figures and images for: Effect of aromatase inhibitors for preventing ovarian hyperstimulation syndrome in infertile patients undergoing in vitro fertilization: a systematic review and meta-analysis
Source: Reprod Biol Endocrinol. 2024 Jul 23;22:85. doi: 10.1186/s12958-024-01258-y (PMC11265326; doi:10.1186/s12958-024-01258-y)

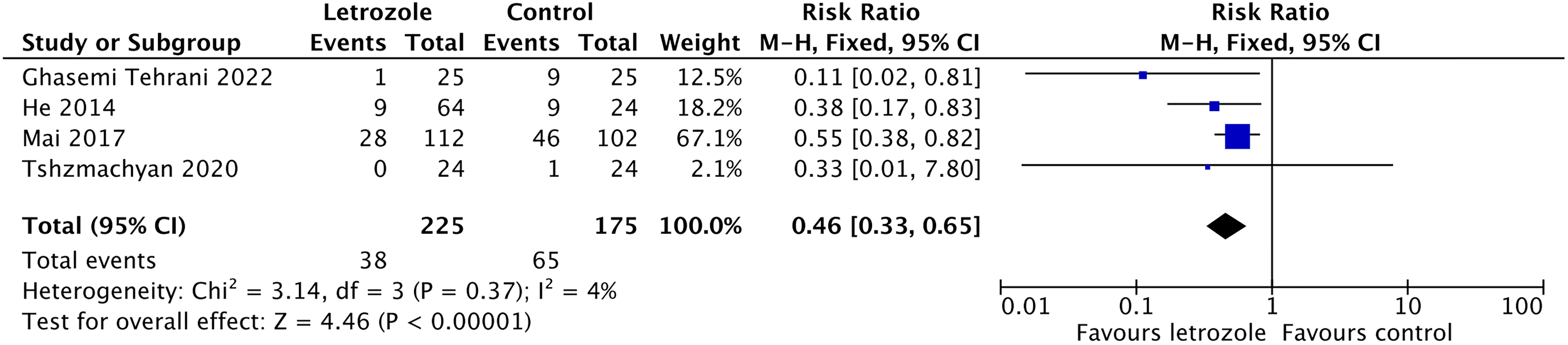

Supplement: Supplementary file 2 — Supplementary Material 2 [file 12958_2024_1258_MOESM2_ESM.png]

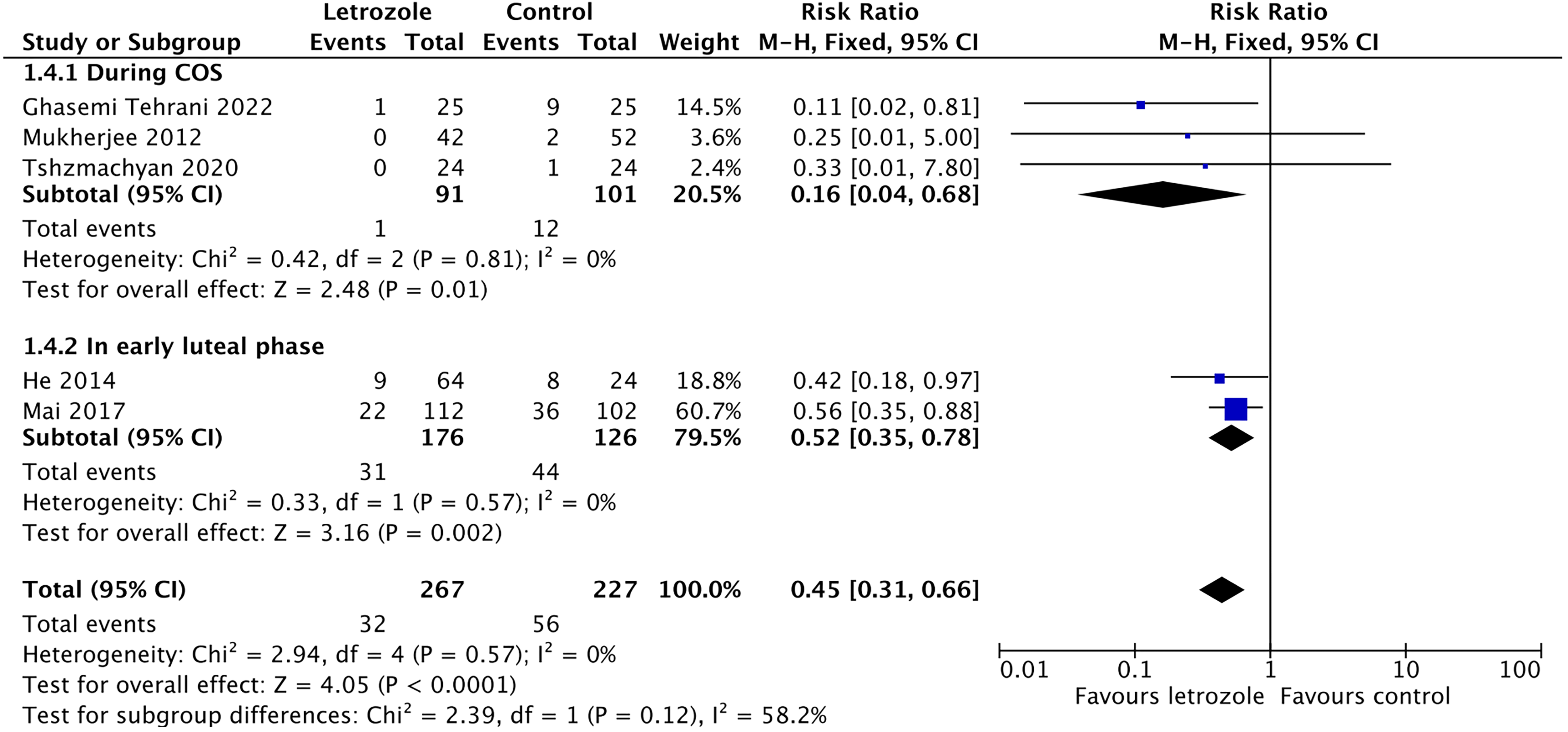

Supplement: Supplementary file 3 — Supplementary Material 3 [file 12958_2024_1258_MOESM3_ESM.png]
